# Irritable bowel syndrome in children with chronic gastrointestinal symptoms in primary care

**DOI:** 10.1093/fampra/cmad070

**Published:** 2023-07-01

**Authors:** Esmee M Hogervorst, Ilse N Ganzevoort, Marjolein Y Berger, Gea A Holtman

**Affiliations:** Department of General Practice, University of Groningen, University Medical Center Groningen, Groningen, The Netherlands; Department of General Practice, University of Groningen, University Medical Center Groningen, Groningen, The Netherlands; Department of General Practice, University of Groningen, University Medical Center Groningen, Groningen, The Netherlands; Department of General Practice, University of Groningen, University Medical Center Groningen, Groningen, The Netherlands

**Keywords:** child, chronic abdominal pain, chronic diarrhoea, irritable bowel syndrome, primary care, prognosis

## Abstract

**Background:**

Irritable bowel syndrome (IBS) is the most common functional gastrointestinal disorder in children. However, in primary care, it is still unknown whether there are differences in the prognosis of children with IBS compared to other diagnostic subgroups. Therefore, our aim was to describe the course of symptoms and health-related quality of life (HRQoL) for children with chronic gastrointestinal symptoms who either do or do not fulfil the Rome criteria for IBS in primary care. Second, we compared the diagnosis of the general practitioner (GP) with the Rome criteria.

**Methods:**

We conducted a prospective cohort study with 1-year follow-up, including children aged 4–18 years with chronic diarrhoea and/or chronic abdominal pain in primary care. During follow-up, the Rome III questionnaire, Child Health Questionnaire, and symptom questionnaires were completed.

**Results:**

A total of 60/104 children (57.7%) fulfilled the Rome criteria for IBS at baseline. Compared to children without IBS, children with IBS were more commonly referred to secondary care, used more laxatives, and more often developed chronic diarrhoea and low physical HRQoL during 1 year. The diagnosis “IBS” from the GP matched the Rome criteria for only 10% of children, as most were diagnosed with “Constipation.”

**Conclusions:**

There seems to be a difference in the treatment and prognosis of symptoms and HRQoL between children with and without IBS in primary care. This suggests that it is relevant to differentiate between these groups. The evaluation and use of feasible criteria to define IBS in different healthcare settings remains subject for further studies.

**Key messages**

- Prognostic differences exist in children with versus without Rome III-based irritable bowel syndrome (IBS).
- Therefore, it is relevant to differentiate between these groups in practice.
- The general practitioner’s diagnosis of IBS matches the Rome criteria for only 10% of children.
- Feasible criteria should be used to define IBS in different healthcare settings.

## Introduction

Chronic gastrointestinal symptoms, such as chronic abdominal pain and chronic diarrhoea, are frequent problems in childhood and therefore constitute a common reason to consult a general practitioner (GP).^1^ In fact, one year after the first visit, nearly half of all children with abdominal pain still suffer from symptoms.^2^ Importantly, older children seem to have a longer duration of abdominal pain compared to younger children.^2^ In approximately 90% of children with chronic gastrointestinal symptoms, the symptoms are functional, indicating that no organic cause for the pain can be found.^3^ Functional gastrointestinal symptoms can have a large impact on the health-related quality of life (HRQoL) of children, as is shown by several studies performed in secondary and tertiary care.^4–6^ In practice, it can be difficult for GPs to predict which children with chronic abdominal complaints will develop persisting symptoms and lower HRQoL and which will not, since literature on the prognosis of these children is limited. Insight in this matter is important, since this can help provide earlier treatment for these children, which may improve their symptoms and quality of life.

In secondary care, the paediatric Rome criteria are used to subdivide several functional gastrointestinal disorders (FGIDs).^7^ The most important FGIDs are irritable bowel syndrome (IBS) and functional abdominal pain (FAP). Of these, IBS is most common with a worldwide pooled prevalence of 8.8%.^8^ Studies performed in tertiary care have shown several differences in the impact of symptoms between children with IBS and FAP. For instance, children with IBS reported lower HRQoL compared to children with FAP.^4,6,9^ Therefore, it seems to be clinically relevant to distinguish between these subgroups in paediatric care.

In primary care, the distinction between IBS and other diagnostic subgroups may be just as relevant. More knowledge about potential differences between these groups could, for instance, help GPs to better predict the prognosis of these children and help determine which type of treatment is best for each patient. For that matter, it is important to verify whether there are indeed differences in the prognosis of these children and whether the diagnosis of the GP corresponds with the diagnosis according to the Rome criteria. Accordingly, this study aims to describe the course of symptoms and HRQoL of children with chronic gastrointestinal symptoms, who either do or do not fulfil the Rome criteria for IBS, during 1-year follow-up in primary care. In addition, we aim to compare the diagnosis of the GP with the Rome criteria and assess the influence of age on the presence of symptoms and HRQoL, since this has been found to be a potentially relevant prognostic factor.^2^

## Material and methods

### Study design and study population

Data were obtained from a prospective cohort study with 1-year follow-up that assessed the diagnostic accuracy of faecal calprotectin for inflammatory bowel disease in children with chronic gastrointestinal symptoms in primary care.^10^ The data were collected between July 2011 and September 2014, and the study was performed at the Department of General Practice at the University Medical Centre Groningen (UMCG). The study was approved by the Medical Ethics Review Committee of the UMCG.

We included children aged 4–18 years presenting with chronic diarrhoea (≥2 weeks or ≥2 episodes in the previous 6 months) and/or chronic abdominal pain (≥2 episodes in the previous 6 months) in primary care. The exclusion criteria were the following: diagnosed with a chronic organic gastrointestinal disorder; complete examination for gastrointestinal symptoms or use of a faecal calprotectin test in the previous 6 months; chronic use (daily use during ≥3 months per year) of antibiotics, non-steroidal anti-inflammatory drugs or oral corticosteroids and problems in understanding the questionnaires.^10^ Children with red flag symptoms were referred to the paediatrician to rule out chronic organic diseases (e.g. inflammatory bowel disease and coeliac disease).

### Study procedure

A total of 38 general practices in the Northern part of the Netherlands agreed to participate in the study. The diagnosis of the GP was extracted from electronic medical records and comprised both the International Classification of Primary Care (ICPC) code and a specified diagnosis. Both at baseline and 12 months follow-up, parents of included children completed 2 questionnaires: the Dutch translation of the Questionnaire on Paediatric Gastrointestinal Symptoms–Rome III version (QPGS-RIII) and the Dutch translation of the Child Health Questionnaire - Parent Form 50 (CHQ-PF50).^11^ In addition, during follow-up, the child (≥10 years) or parent completed a symptom questionnaire every 3 months (baseline, 3, 6, 9, and 12 months).

### Questionnaires

#### Questionnaire on Paediatric Gastrointestinal Symptoms–Rome III version (QPGS-RIII).

 The QPGS-RIII is a validated diagnostic questionnaire that assesses the presence of each of the 10 FGIDs, based on the Child/Adolescent Rome III criteria for FGIDs^7,12,13^ (**Supplementary Material 1**). Accordingly, the QPGS-RIII consists of 62 questions about gastrointestinal symptoms that correspond with the Rome III criteria of each FGID, including IBS and FAP. During the time of this study’s conduction, the Rome IV criteria had not yet been developed.

#### Symptom questionnaire.

A study-specific symptom questionnaire for use during 12 months follow-up was designed in close collaboration with GPs and paediatric gastroenterologists. These questionnaires consisted of questions about, amongst others, health care visits and the presence of chronic diarrhoea and chronic abdominal pain, according to the definition used in the inclusion criteria, in the previous 3 (instead of 6) months.^14^

#### Child Health Questionnaire - Parent Form 50 (CHQ-PF50).

 The CHQ-PF50 is a validated questionnaire, consisting of 50 items divided into 15 scales, that assesses the parent-reported HRQoL of children.^11^ Scale-specific scores were calculated and combined, providing an overall Physical Summary Score (PhS) and Psychosocial Summary Score (PsS) (**Supplementary Material 2**). The PhS and PsS scores describe the overall quality of the parent-reported physical and psychosocial health of the child, respectively. The score range for the individual scale scores and for the PhS and PsS is 0–100, with 0 being the lowest possible HRQoL and 100 being the highest. Low physical and/or psychosocial HRQoL was defined as having a PhS and/or PsS score below a norm score of 50, respectively.^11^

### Statistical analyses

For statistical analyses, the total group was divided into children with and without IBS, based on whether they, respectively, did or did not fulfil the Rome III criteria for IBS at baseline. Descriptive statistics were used to give an overview of the course of symptoms (chronic abdominal pain, chronic diarrhoea) and HRQoL scores of children with and without IBS during 12 months follow-up. We also used descriptive statistics to compare the diagnosis of the GP with the Rome criteria for both groups and to describe the influence of age on the presence of symptoms and HRQoL. For the latter, children in both groups were divided into the categories young (4–9 years) and old (10–18 years). IBM SPSS Statistics for Windows, version 25.0.0.2 (IBM Corp., Armonk, NY, USA) was used for statistical analyses.

## Results

### Baseline characteristics

The total patient group consisted of 114 children. Of these, 10 children were excluded as it could not be established whether they had IBS, due to missing data from the QPGS-RIII at baseline. These children had similar proportions of symptoms compared to included children and none of these children received the diagnosis IBS from their GP.

Of the remaining 104 children, the median age was 9.0 years (6.0–12.0), and 66.3% were girls (**Table 1**). A total of 60 children (57.7%) fulfilled the Rome criteria for IBS at baseline. These children more often had red flag symptoms at baseline, were more commonly referred to secondary care, and used more laxatives compared to the 44 children without IBS (**Table 1**). Four children were diagnosed with an organic disease at baseline: 3 children had a positive faeces test for Giardia Lamblia, and 1 tested positive for Shiga toxin-producing *Escherichia coli*.

**Table 1.** Baseline characteristics of the total group and children with and without irritable bowel syndrome (*N =* 104), 2011–2014

Characteristics	Total group (*N* = 104)	IBS (*n* = 60)	No IBS (*n* = 44)
Age in years, median (IQR)	9.0	9.0	9.0
	(6.0–12.0)	(6.0–12.0)	(6.0–12.75)
Age category, *n* (%)			
4–9 years	58 (55.8)	35 (58.3)	23 (52.3)
10–18 years	46 (44.2)	25 (41.7)	21 (47.7)
Sex, *n* (%)			
Boys	35 (33.7)	19 (31.7)	16 (36.4)
Girls	69 (66.3)	41 (68.3)	28 (63.6)
Consult general practitioner in past^a^, *n* (%)	79 (76.0)	45 (75.0)	34 (77.3)
Consult paediatrician in past^b^, *n* (%)	36 (34.6)	23 (38.3)	13 (29.5)
≥1 red flag symptom according to general practitioner, *n* (%)	34 (32.7)	22 (36.7)	12 (27.3)
Referral to secondary care, *n* (%)	28 (26.9)	20 (33.3)	8 (18.2)
Medication use, *n* (%)			
Laxatives (total)^c^	37 (35.6)	25 (41.7)	13 (29.5)
Nonspecified	5 (4.8)	4 (6.7)	1 (2.3)
Macrogols	26 (25.0)	18 (30.0)	9 (20.5)
Lactulose	6 (5.8)	3 (5.0)	3 (6.8)
Anti diarrhoeals	2 (1.9)	2 (3.3)	0 (0.0)
Anti spasmodics	1 (1.0)	1 (1.7)	0 (0.0)

^a^Consultation at any general practitioner for chronic abdominal complaints in the past.

^b^Consultation at any paediatrician for chronic abdominal complaints in the past.

^c^Daily use of laxatives for a period shorter than 3 months.

### Course of the diagnosis

Of the 60 children with IBS at baseline, only 12 (22.2%) still fulfilled the criteria for IBS after 12 months (**Table 2**). Of the 44 children without Rome III-based IBS, 31 children (70.5%) fulfilled the criteria for an FGID at baseline, of whom 9 had functional constipation. After 12 months, only 12 children (27.3%) in this group still fulfilled the criteria for an FGID, of whom two fulfilled the criteria for IBS. In both groups, aerophagia was common.

**Table 2.** Other functional gastrointestinal disorders according to the Rome III criteria at baseline and 12 months follow-up in children with and without irritable bowel syndrome

	IBS		No IBS	
Rome III criteria	Baseline (*n* = 60)	12 months (*n* = 54)	Baseline (*n* = 44)	12 months (*n* = 36)
IBS	60 (100.0)	12 (22.2)	0 (0.0)	2 (5.6)
Abdominal migraine	18 (31.0)	10 (18.9)	10 (22.7)	3 (8.6)
Aerophagia	27 (46.6)	14 (25.9)	13 (29.5)	6 (16.7)
Functional abdominal pain syndrome	0 (0.0)	1 (1.9)	5 (11.4)	1 (2.8)
Functional constipation	0 (0.0)	2 (3.7)	9 (20.5)	2 (5.6)
Other^a^	3 (5.0)	1 (1.9)	5 (11.4)	1 (2.8)

^a^Other functional gastrointestinal disorders: functional dyspepsia, cyclic vomiting syndrome, rumination syndrome.

At baseline, only 6 children (10.0%) with Rome III-based IBS received the same diagnosis from their GP; instead, in both groups, most children received the diagnosis “Constipation” and “Non-specified abdominal complaints” (**Table 3**). In the group of children without Rome III-based IBS, 3 children were diagnosed with IBS by their GP, whereas they fulfilled the Rome criteria for aerophagia.

**Table 3.** Diagnosis of the general practitioner for children with and without irritable bowel syndrome according to the Rome III criteria at baseline

Diagnosis GP	IBS (*n* = 60)	No IBS (*n* = 44)
ICPC-code GP at baseline, *n* (%)		
Generalized abdominal pain (D01)	13 (21.7)	16 (36.4)
Gastric pain (D02)	1 (1.7)	0 (0.0)
Otherwise localized abdominal pain (D06)	7 (11.7)	10 (22.7)
Constipation (D12)	13 (21.7)	3 (6.8)
Irritable bowel syndrome (D93)	5 (8.3)	2 (4.5)
Urinary tract infection (U71)	1 (1.7)	0 (0.0)
Other ICPC code	0 (0.0)	2 (4.5)
No ICPC code	20 (33.3)	11 (25.0)
Specified diagnosis GP at baseline, *n* (%)		
Irritable bowel syndrome	6 (10.0)	3 (6.8)
Constipation with or without abdominal pain	20 (33.3)	11 (25.0)
Functional abdominal pain	2 (3.3)	7 (15.9)
(Previous) gastroenteritis	3 (5.0)	2 (4.5)
Suspected urinary tract infection	1 (1.7)	1 (2.3)
Suspected food allergy	3 (5.0)	0 (0.0)
Non-specified (chronic) abdominal complaints	21 (35.0)	19 (43.2)
Other	4 (6.7)	1 (2.3)

### Course of symptoms

**Fig. 1.**
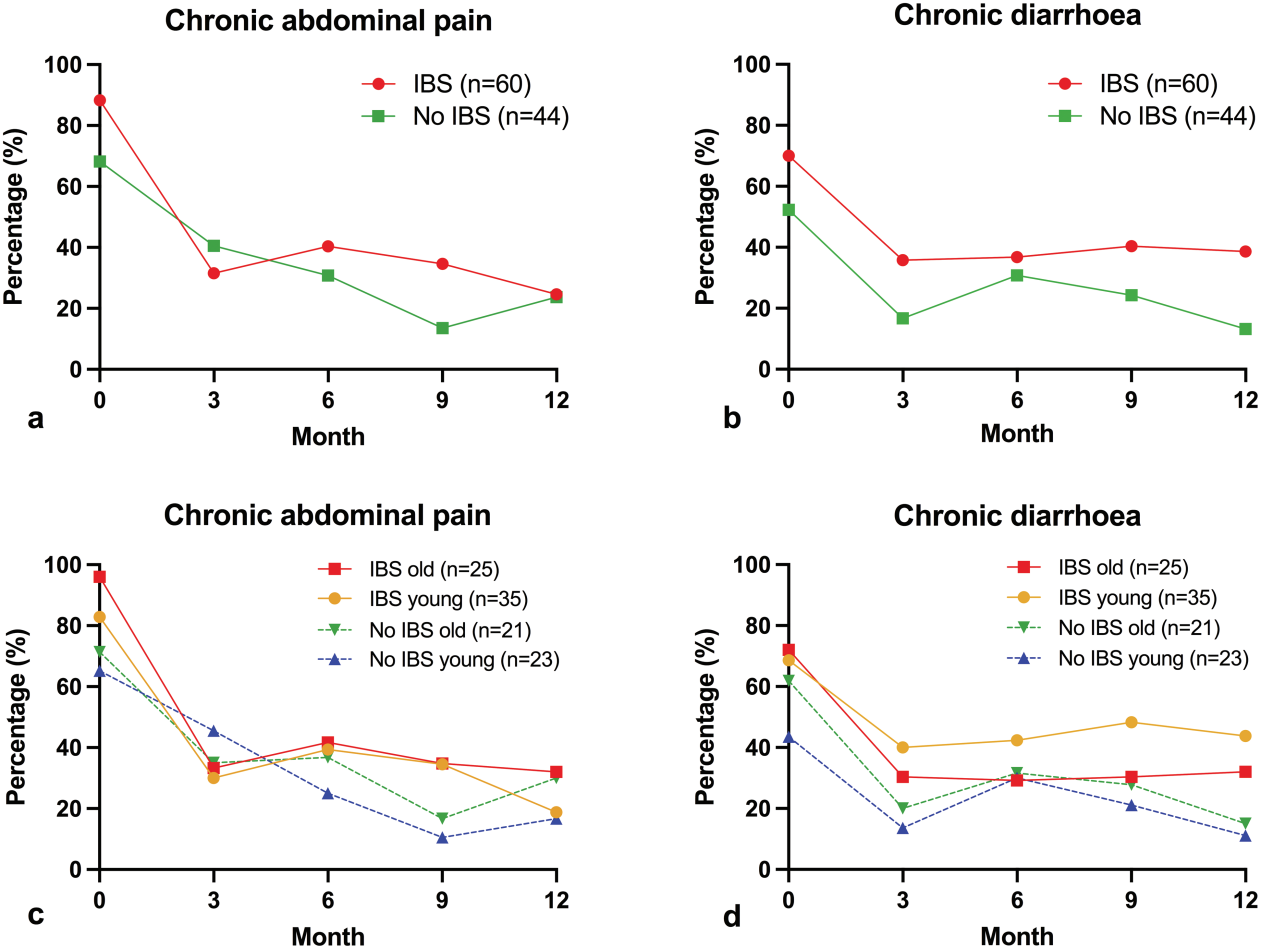
Twelve-month course of the percentage of a) chronic abdominal pain in children with and without irritable bowel syndrome; b) chronic diarrhoea in children with and without irritable bowel syndrome; c) chronic abdominal pain in young and old children in both groups; d) chronic diarrhoea in young and old children in both groups.

For children with and without IBS, percentages of chronic abdominal pain and chronic diarrhoea were highest at baseline (88.3% and 70.0% vs. 68.2% and 52.3%, respectively), decreased by (more than) half within the first 3 months and approximately stabilized thereafter (**Fig. 1**). After 1 year, respectively 24.6% and 38.6% of children with IBS still had chronic abdominal pain and chronic diarrhoea, compared to 23.7% and 13.2% of children without IBS. At each follow-up moment, percentages of chronic diarrhoea were highest for children with IBS, compared to children without IBS. For chronic abdominal pain, these differences were less marked. The course of symptoms was similar for young and older children in both groups (**Fig. 1**). After 12 months, 9 children (8.7%) did not complete the symptom questionnaire. They did not differ from the other children as regards sex, age, and the presence of chronic abdominal pain or chronic diarrhoea at baseline.

### Course of HRQoL

In both children with and without IBS, proportions of low physical and psychosocial HRQoL were highest at baseline compared to 12 months (**Table 4**). At baseline, children in both groups more often demonstrated low physical HRQoL than low psychosocial HRQoL.

**Table 4.** Health-related quality of life perception based on Physical and Psychosocial Summary Scale scores in children with or without irritable bowel syndrome

	IBS		No IBS	
CHQ-PF50 scale scores	Baseline (*n* = 55)	12 months (*n* = 50)	Baseline (*n* = 39)	12 months (*n* = 33)
Physical Summary Scale score <50, *n* (%)				
All ages	33 (60.0)	11 (22.0)	18 (46.2)	10 (30.3)
Per age category				
Young (4–9 years)	18 (51.4)	6 (20.7)	7 (38.9)	3 (17.6)
Old (10–18 years)	15 (75.0)	5 (23.8)	11 (52.4)	7 (43.8)
Psychosocial Summary Scale score <50, *n* (%)				
All ages	20 (36.4)	15 (30.0)	11 (28.2)	7 (21.2)
Per age category				
Young (4–9 years)	13 (37.1)	8 (27.6)	5 (27.8)	3 (17.6)
Old (10–18 years)	7 (35.0)	7 (33.3)	6 (28.6)	4 (25.0)

During follow-up, physical HRQoL increased more than psychosocial HRQoL. Overall, at each follow-up moment, children with IBS showed greater percentages of low physical and psychosocial HRQoL than children without IBS, except for physical HRQoL at 12 months.

Regarding the influence of age, in both diagnostic groups, older children more often experienced low physical HRQoL than young children, both at baseline and 12 months (**Table 4**). For psychosocial HRQoL, differences between age groups were limited.

## Discussion

Our study demonstrates that children with IBS are more commonly referred to secondary care, use more laxatives and more often develop chronic diarrhoea and low physical HRQoL during 1 year compared to children without IBS. The course of chronic abdominal pain was relatively similar for both groups. After 1 year, approximately 25% and 40% of children with IBS still have chronic abdominal pain and chronic diarrhoea, compared to 23.7% and 13.2% in children without IBS. Remarkable is that the diagnosis “IBS” from the GP matches the Rome criteria for only 10% of children, whereas these children most often receive the diagnosis “Constipation.” In both diagnostic groups, older children more often experienced low physical HRQoL than young children.

### Comparison with literature

Our finding that children with IBS seem to have a similar prognosis in terms of abdominal pain than children without IBS differs from the results of Lisman-van Leeuwen et al.^2^ This primary care-based prospective cohort study demonstrated that children with symptoms resembling IBS had a longer duration of chronic abdominal pain than children with symptoms of FAP or functional dyspepsia (9.0 months compared to 7.5 months, respectively).^2^ Our finding that children with IBS seem to have a less favourable prognosis in terms of chronic diarrhoea and HRQoL compared to children without IBS has not been found before. Although our study is small and the clinical relevance of these findings should be further evaluated, our findings in combination with earlier studies indicate that it could be relevant for GPs to make a diagnostic distinction between children with and without IBS.

In terms of HRQoL, our study shows that children with and without IBS mostly suffer from a decrease in physical HRQoL, rather than psychosocial HRQoL. The fact that only a minority of children in our study experienced reduced psychosocial HRQoL is striking, since literature in tertiary care showed that FGIDs have a negative impact on the HRQoL of children across all dimensions, including emotional and social domains.^4^ This discrepancy could be due to the fact that children in tertiary care are reported to have more severe symptoms than children in community care.^15^

Finally, in our study, older children more frequently suffered from chronic abdominal pain and chronic diarrhoea during follow-up and experienced lower physical HRQoL compared to younger children. These results correspond with findings from Spee et al.,^16^ a prospective cohort study in primary care, which showed a positive association between older age and the presence of chronic abdominal pain at 1 year. An explanation for these findings could be that adolescent children (10–18 years) are in a different developmental phase compared to younger children (4–9 years), which is associated with multiple physical and psychological changes that may negatively influence the perception of abdominal pain and the impact on HRQoL.^17^ These findings demonstrate the importance for GPs to be extra alert on persistence of symptoms and decrease in HRQoL in older children.

### Strengths and limitations

Whereas previous primary care-based studies have mostly focused on the prognosis of abdominal pain in children with chronic gastrointestinal symptoms, our study also describes the course of chronic diarrhoea and HRQoL. In addition, by making use of the QPGS-RIII, we utilized the official Rome criteria to divide children into diagnostic subgroups, which previous primary care-based studies have not done. However, since our data are rather dated, we made use of the Rome III criteria instead of the currently most recent Rome IV criteria. Differences between the Rome III and Rome IV criteria of IBS include the removal of the term “abdominal discomfort” and the change of the duration criterium from “at least once per week” to “at least four days per month.” These changes in the Rome IV criteria have reportedly resulted into twice as many diagnoses of IBS.^18^ Accordingly, in our study, more children may have fulfilled the criteria of IBS using the Rome IV criteria instead of the Rome III criteria.

However, it is unknown whether these children would receive the same diagnosis from their GP. Therefore, the numbers found in our descriptive study could be somewhat under- or overestimated compared to new criteria. Despite the use of dated Rome criteria, our study is the first to give insight into the prognostic differences between children with and without IBS in primary care. Our findings, therefore, provide useful knowledge for health practitioners and form an important base for future studies with higher sample size, which should verify our findings and the effect of different diagnostic criteria.

For the definition of chronic diarrhoea and chronic abdominal pain, we did not use validated questionnaires. Instead, these correspond with the guidelines of the Dutch College of General Practitioners. A limitation of our study is the relatively small sample size, which refrained us from using inferential statistics. Therefore, our results are subject to a notable degree of uncertainty. Furthermore, studies with larger sample sizes could compare the prognosis between children with different FGIDs in primary care.

## Conclusion and clinical implications

Based on our results, there seems to be a difference in the prognosis of symptoms and HRQoL and treatment (i.e. use of laxatives and referral to secondary care) between children with chronic gastrointestinal symptoms who either do or do not fulfil the Rome criteria for IBS. This suggests that it is relevant to differentiate between these groups of children in daily practice. However, our study also shows that only 10% of children with Rome III-based IBS receive the same diagnosis from the GP, which illustrates that Dutch GPs infrequently use these criteria in practice. Infrequent use of the Rome III criteria by GPs can be explained by the fact that the Rome criteria are not commonly known in primary care and that it takes relatively much time to use them in practice, due to their extensiveness. Instead, Dutch GPs make use of the definition of IBS from the guidelines of the Dutch College of General Practitioners: “(recurrent) episodes of abdominal pain or abdominal discomfort, associated with changes in stool (frequency and/or form)”.^19^ As a potential consequence, under the assumption that paediatricians more commonly use the Rome criteria in practice, GPs may classify children with IBS differently compared to paediatricians. To further evaluate and fully understand prognosis in relation to symptomatic criteria, it is important that primary care physicians and paediatricians in secondary care and further, are aware of their diagnostic criteria and criteria to start treatment. Ideally, these criteria are the same for all settings, in order to improve intercollegiate communication regarding these children. The evaluation and use of feasible criteria to define IBS in different health care settings remains subject for further studies.

## Supplementary material

Supplementary material is available at *Family Practice* online.

cmad070_suppl_Supplementary_Material_1

cmad070_suppl_Supplementary_Material_2

cmad070_suppl_Supplementary_Checklist

## Data Availability

The data underlying this article are available on request from the corresponding author, GAH. The data are not publicly available due to privacy and ethical concerns.
