# Supplementary material for: Irritable bowel syndrome in children with chronic gastrointestinal symptoms in primary care
Source: Fam Pract. 2023 Jul 1;41(3):292–8. doi: 10.1093/fampra/cmad070 (PMC11167984; doi:10.1093/fampra/cmad070)
Supplement: cmad070_suppl_Supplementary_Material_1 [file cmad070_suppl_supplementary_material_1.docx]

**Supplementary 1: Rome III criteria for functional gastrointestinal disorders in children or adolescents *(content obtained from Rasquin et al. (7))***

| **FGID** | **Rome III criteria** |
| --- | --- |
| **Abdominal-pain related FGIDs** | |
| **Abdominal migraine** | All of:  1. Paroxysmal episodes of intense, acute umbilical pain that last ≥ 1 hour  2. Intervening periods of usual health that last weeks to months  3. The pain interferes with normal activities  4. The pain is associated with ≥2 of:  a. Anorexia  b. Nausea  c. Vomiting  d. Headache  e. Photophobia  f. Pallor  5. No evidence of an inflammatory, anatomic, metabolic or neoplastic process that explains the symptoms  *Duration criterium: must have fulfilled criteria ≥2 times in the previous 12 months* |
| **Childhood functional abdominal pain** | All of:  1. Episodic or continuous abdominal pain  2. Insufficient criteria for other FGIDs  3. No evidence of an inflammatory, anatomic, metabolic or neoplastic process that explains the symptoms  *Duration criterium: must have fulfilled criteria at least once per week for ≥2 months prior to diagnosis* |
| **Childhood functional abdominal pain syndrome** | Fulfils criteria of childhood FAP and has ≥25% of the time ≥1 of:  1. Some loss of daily functioning  2. Additional somatic symptoms such as headache, limb pain or difficulty sleeping  *Duration criterium: must have fulfilled criteria at least once per week for ≥2 months prior to diagnosis* |
| **Functional dyspepsia** | All of:  1. Persistent or recurrent pain or discomfort centred in the upper abdomen (above the umbilicus)  2. Not relieved by defecation or associated with the onset of a change in stool frequency or stool form (i.e. not IBS)  3. No evidence of an inflammatory, anatomic, metabolic or neoplastic process that explains the symptoms  *Duration criterium: must have fulfilled criteria at least once per week for ≥2 months prior to diagnosis* |
| **Irritable bowel syndrome** | Both of:  1. Abdominal discomfort^1^ or pain associated with (≥25% of the time) ≥2 of:  a. Improvement with defecation  b. Onset is associated with a change in frequency of stool  c. Onset is associated with a change in form (appearance) of stool  2. No evidence of an inflammatory, anatomic, metabolic or neoplastic process that explains the symptoms  *Duration criterium: must have fulfilled criteria at least once per week for ≥2 months prior to diagnosis* |
| **Constipation and incontinence**  **Supplementary 1: Rome III criteria for functional gastrointestinal disorders in children**  **or adolescents *(content obtained from Rasquin et al. (7))*** | |
| **Functional constipation** | In a child with a developmental age of ≥4 years with insufficient criteria for a diagnosis of IBS, ≥2 of:  1. ≤2 defecations in the toilet per week  2. ≥1 episode of fecal incontinence per week  3. History of retentive posturing or excessive volitional stool retention  4. History of painful or hard bowel movements  5. Presence of a large faecal mass in the rectum  6. History of large diameter stools that may obstruct the toilet  *Duration criterium: must have fulfilled criteria at least once per week for ≥2 months prior to diagnosis* |
| **Non-retentive faecal incontinence** | In a child with a developmental age of ≥4 years, all of:  1. Defecation into places inappropriate to the social context at least once per month  2. No evidence of an inflammatory, anatomic, metabolic or neoplastic process that explains the symptoms  3. No evidence of faecal retention  *Duration criterium: must have fulfilled criteria for ≥2 months prior to diagnosis* |
| **Vomiting and aerophagia** | |
| **Adolescent rumination syndrome** | All of:  1. Repeated painless regurgitation and rechewing or expulsion of food that  a. Begin soon after ingestion of a meal  b. Do not occur during sleep  c. Do not respond to standard treatment for gastroesophageal reflux2. No retching  3. No evidence of an inflammatory, anatomic, metabolic or neoplastic process that explains the symptoms  *Duration criterium: must have fulfilled criteria at least once per week for ≥2 months prior to diagnosis* |
| **Aerophagia** | ≥2 of:  1. Air swallowing  2. Abdominal distention due to intraluminal air  3. Repetitive belching and/or increased flatus  *Duration criterium: must have fulfilled criteria at least once per week for ≥2 months prior to diagnosis* |
| **Cyclic vomiting syndrome** | Both of:  1. ≥2 periods of intense nausea and unremitting vomiting or retching that last hours to days  2. Return to usual health status that lasts weeks to months |

FAP: functional abdominal pain; FGID: functional gastrointestinal disorder; IBS: irritable bowel syndrome.

**Supplementary 1: Rome III criteria for functional gastrointestinal disorders in children**

**or adolescents *(content obtained from Rasquin et al. (7))***

^1^an uncomfortable sensation that is not described as pain
