# Supplementary material for: Irritable bowel syndrome in children with chronic gastrointestinal symptoms in primary care
Source: Fam Pract. 2023 Jul 1;41(3):292–8. doi: 10.1093/fampra/cmad070 (PMC11167984; doi:10.1093/fampra/cmad070)
Supplement: cmad070_suppl_Supplementary_Material_2 [file cmad070_suppl_supplementary_material_2.docx]

**Supplementary 2: Child Health Questionnaire - Parent Form 50 scales and scoring methods (*adapted from the HealthActCHQ manual (12))***

| **CHQ-PF50 scale** | **Description** | **Number of items** |
| --- | --- | --- |
| Global health | Measures the parent-reported assessment of the child’s general health | 1 |
| Physical functioning | Measures the parent-reported physical limitations that the child experiences due to problems in health | 6 |
| Role/social limitations – emotional/behavioral | Measures the parent-reported school-based and social limitations that the child experiences due to emotional behavioral problems | 3 |
| Role/social limitations – physical | Measures the parent-reported school-based and social limitations that the child experiences due to physical problems in health | 2 |
| Bodily pain/discomfort | Measures the parent-reported intensity and frequency of bodily pain or discomfort in the child | 2 |
| General behavior (scales: Behaviour and Global Behaviour item) | Measures the parent-reported intensity and frequency of behavioral problems in the child | 6 |
| Mental health | Measures the parent-reported frequency of an anxious, depressed and positive state of mind in the child | 5 |
| Self esteem | Measures the parent-reported satisfaction of the child with his/her looks, abilities in school and sports, bond with family members and others, and with life overall | 6 |
| General health perceptions | Measures the parent-reported health of the child in the past, present and future, and the child’s susceptibility to illness | 5 |
| Change in health | Measures the parent-reported change in health of the child over the previous 12 months | 1 |
| Parental impact – emotional | Measures the intensity of distress and worry that the parent experiences regarding the child’s problems in health, behaviour, state of mind, or learning | 3 |
| Parental impact – time | Measures the intensity of limitations in personal time that the parent experiences due to the child’s problems in health, behaviour, state of mind, or learning | 3 |
| Family activities | Measures the parent-reported intensity of limitations or disturbance in family activities due to the child’s problems in health or behaviour | 6 |
| Family cohesion | Measures the parent-reported assessment of the relationship between family members | 1 |

CHQ-PF50: Child Health Questionnaire-Parent Form 50

**Calculation of CHQ-PF50 scores:**

1. Check whether the answers on all items are within the correct range. If not, convert the answer to missing.

2. The highest score of each item should indicate better health. Recalibrate, therefore, the answers on items of the following scales: Global Health, Bodily pain/discomfort, Global behaviour item, Mental Health scale item 6.1e, Self esteem, General health perceptions items 8.1b and 8.1d, Change in health, Parental impact-emotional, Family cohesion.

3. Count the number of completed items for each scale.

4. Scale scores are imputed for any participant who has completed ≥50% of the items in the

scale, using the formula: $Actual Raw Score=\frac{sum of item responses}{number of completed items}$. Convert the scale score to

missing for participants who have completed <50% of the items.

5. Transform the Actual raw score of each scale to a 0-100 range by calculating the Transformed

Raw Score, using the scale-specific formula:

$Transformed Raw Score=\frac{Actual Raw Score-Lowest possible Raw Score}{Possible Raw Score range}$ *x 100*. The possible raw

score range is defined as the highest possible raw score minus the lowest.

**Calculation of PhS and PsS scores:**

The Physical Summary (PhS) and Psychosocial Summary (PsS) scores describe the overall

physical and psychosocial health status of the child. These can both be calculated from the scores of

the 10 following scales: Physical functioning, Role/social limitations – emotional/behavioural, Role/social limitations – physical, Bodily pain/discomfort, Behaviour, Mental health, Self esteem, General Health perceptions, Parental impact – time, Parental impact – emotional.

1. Check whether the scale scores have been correctly transformed.

2. Transform each scale score into a z-score (standardization) by substracting the general U.S.

population mean from the scale score. Divide the outcome by the general U.S. population

standard deviation.

3. Calculate an aggregate raw PhS score by multiplying the z-score of each scale by the

corresponding physical factor score coefficient (from the general U.S. population and six

clinical samples) and sum up all 10 outcomes. The same is done for the raw PsS score, although

each z-score should now be multiplied by the psychosocial factor score coefficient. Convert the

raw PhS and PsS score to missing if any of the ten scale scores is missing.

4. Transform the raw PhS and PsS scores to a norm-based score by multiplying both scores by

10 and adding 50 to the outcome.
